# Three-Year Changes in Visual Function in the Placebo Group of a Randomized Double-Blind International Multicenter Safety Study: Analysis of Electroretinography, Perimetry, Color Vision, and Visual Acuity in Individuals With Chronic Stable Angina Pectoris

**DOI:** 10.1167/tvst.11.1.2

**Published:** 2022-01-04

**Authors:** Eberhart Zrenner, Graham E. Holder, Ulrich Schiefer, John M. Wild

**Affiliations:** 1 Center for Ophthalmology, University of Tübingen, Tübingen, Germany; 2 Werner Reichardt Center for Integrative Neuroscience (CIN), University of Tübingen, Tübingen, Germany; 3 Moorfields Eye Hospital, London, UK; 4 University College London, Institute of Ophthalmology, London, UK; 5 Department of Ophthalmology, Yong Loo Lin School of Medicine, National University of Singapore, Singapore; 6 Competence Center Vision Research, University of Applied Sciences Aalen, Aalen, Germany; 7 College of Biomedical and Life Sciences, Cardiff University, Cardiff, UK

**Keywords:** aging, visual function, clinical trial, best-corrected visual acuity, color vision, electroretinography, static automated perimetry (SAP), semi-automated kinetic perimetry (SKP), correction factor for aging

## Abstract

**Purpose:**

To determine whether significant deteriorations in objective (electroretinography [ERG]) and subjective (standard automated and semi-automated kinetic perimetry; color discrimination; and best-corrected visual acuity) tests of visual function, potentially attributable to aging, occurred in the group randomized to placebo of a 3-year prospective multicenter ocular safety study of ivabradine for chronic stable angina pectoris.

**Methods:**

The multicenter trial was conducted at 11 international ophthalmic centers. Changes in visual function between baseline and month 36 were analyzed by means of a two-tailed Wilcoxon signed-rank test, based on the Hodges and Lehman estimator of the median difference, with the 95% confidence intervals derived by Walsh averages.

**Results:**

Thirty-eight participants from the placebo group completed the study (mean [SD], age, 62.7 [8.1] years). The group exhibited in each eye small, but statistically significant, reductions in the amplitudes of the dark-adapted (DA) ERG 3.0 a-wave, and light-adapted (LA) 3.0 b-wave, as well as increases in peak time for the DA 0.01 b-wave, DA 3.0 a-wave, LA 3.0 b-wave, and LA 3.0 30-Hz flicker response and in the isopter area I3e of the visual field.

**Conclusions:**

Statistically significant deteriorations occurred in visual function over a period of 3 years, potentially attributable to age, within a group of individuals with chronic stable angina pectoris and unremarkable ophthalmological findings other than those attributable to age.

**Translational Relevance:**

A longitudinal correction factor for age-related change in visual function may be useful in future trials to determine whether an observed deterioration in visual function is related to intervention or to aging.

## Introduction

When assessing safety in longitudinal prospective clinical trials of therapeutic interventions for slowly progressive retinal disorders, it is important to separate treatment effects from those due to physiologic age-related changes. Age-related effects have been reported for tests of visual function, including high-contrast best-corrected visual acuity (BCVA),^1^^–^^6^ color vision (CVis) arrangement tests,^7^ electroretinography (ERG),^8^^–^^11^ standard automated perimetry (SAP),^12^^–^^18^ and semi-automated kinetic perimetry (SKP).^19^^,^^20^ Such outcomes are usually based on cross-sectional data collection at a given time period for a given individual of a given age, which is then evaluated with those from other individuals of differing ages within the same study. Other studies have evaluated age-related change on a longitudinal basis by comparing the outcome from an individual at baseline with that derived for the same individual on a subsequent occasion after a given time interval. The “cross-sectional” method is considered a less elegant/satisfactory indicator than that determined by longitudinal studies not only for ocular functional^8^^–^^10^ and structural outcomes^21^^–^^23^ but also for other tissues such as muscle function.^24^ Such studies, whether cross-sectional or longitudinal, are usually undertaken on ophthalmologically and systemically “normal” individuals. Very few, if any, studies of aging have been undertaken on cohorts with a specific ocular or systemic disease.

We have previously reported an age-related cross-sectional decline (<60 years of age at baseline compared with 60–79 and ≥70 years of age) in each of the 5 International Society for Clinical Electrophysiology of Vision (ISCEV), standard ERGs (standard^25^ applicable at study conception), for the Lanthony desaturated D15 color vision arrangement test, for SAP, and for the I3e and the III4e isopter areas obtained by SKP.^26^ These findings were derived from a 3-year multicenter, international, prospective, double-blind, randomized placebo-controlled safety study of ivabradine, a selective inhibitor of the cardiac pacemaker current (I_f_), in participants with chronic stable angina pectoris who were receiving standard background anti-angina therapy (EudraCT No. 2006-005475-17). The primary objective of the trial, which was requested by the European Medicines Agency (EMA), was to “document further the long-term ocular safety of ivabradine.” The primary endpoint was to assess by ERG the expected pharmacologic effect, as well as its potential reversibility, of the I_f_ inhibitor on the corresponding retinal current, I_h_, generated by the hyperpolarization-activated cyclic nucleotide-gated cation channels.^27^

The trial involved 97 participants in 19 cardiology centers and 11 associated ophthalmologic centers in 9 countries in Europe, Asia, South America, and Australia and used standardized equipment, stringent quality-controlled procedures, and centrally monitored procedures.^26^

The duration of phase III and IV safety and efficacy clinical trials varies with the therapy and, therefore, with the disease entity in question. Given the cross-sectional evidence of an age-related effect on visual function, it would seem sensible to investigate whether a longitudinal age effect could be detected over the typical 2- to 4-year period/timeframe in multicenter studies associated with, or involving, chronic primary or secondary ocular disease or possible ophthalmic adverse events. Such an age effect, if present, would become an important analytical factor in future studies, of similar or longer duration, designed to examine the safety and efficacy of a treatment intervention, especially if a control group is not used in phase IV.

This study examines the effects of age on visual function, studied longitudinally over 3 years, in the randomized-to-placebo group of individuals with stable chronic angina pectoris and unremarkable ophthalmologic findings, other than those attributable to age, who participated in the multicenter trial of ivabradine.^26^

## Methods

### Study Design and Cohort

The methodology has been reported in full elsewhere.^26^ The 11 ophthalmologic centers were specialized in both ERG and perimetry. Testing equipment and methodology were identical across all centers. Prior to the onset of the study, the local ophthalmological investigator and the ophthalmic technicians at each of the centers received 2 days of expert face-to-face hands-on instruction in the study protocol, including the uploading of the data into the central database. Each center was then required to complete a certification procedure for both ERG and perimetry, to the satisfaction of the respective experts of the Scientific Ophthalmic Safety Committee who were masked to the center.

Potential participants initially attended pre-inclusion visits for cardiovascular and full ophthalmologic examinations, including high-contrast BCVA, CVis arrangement testing, SAP, SKP, tonometry, ERG recording, and fundus photography. Those who met the cardiac, medical, and ophthalmologic inclusion criteria but who failed to achieve a satisfactory ERG recording were given one further opportunity to produce an acceptable recording at the first of two pre-inclusion visits undertaken within 5 days of the initial ophthalmologic visit. The color vision testing and both types of perimetric tests were repeated for all potential participants at the first pre-inclusion visit to reduce the impact of learning effects.^28^^,^^29^ Those who failed to provide a reliable result at the first pre-inclusion visit for either or both SAP and SKP underwent a final attempt, within 5 days, at the second pre-inclusion visit.

Chronic stable angina pectoris was defined as at least one attack of angina per month for the preceding 3 months prior to pre-selection and no angina attack at rest. General exclusion criteria comprised an age of less than 18 years, severe hepatic impairment (three times the normal value for age), renal insufficiency (serum creatinine >200 µmol/L), electrolyte disorders, and thyroid abnormalities unless stable and controlled by thyroxine for at least 3 months prior to pre-selection. Additional exclusion criteria involving markers for previous or current systemic disease and previous or current comedications are given in Annex A of Supplemental Material.

Ophthalmic exclusion criteria comprised a BCVA of worse than 0.5 (decimal), myopia or hypermetropia of >5.00 Diopters Sphere (DS), astigmatism >3.00 Diopters Cylinder (DC) in either eye, nystagmus, impending cataract surgery, an intraocular pressure >25 mm Hg if associated with an optic nerve head appearance, and/or a visual field defect, characteristic of glaucoma, angle closure glaucoma, chronic inflammatory eye disease, proliferative diabetic retinopathy, nonproliferative diabetic retinopathy with more than five microaneurysms and/or hard exudates and/or intraretinal hemorrhages, macular edema or progressive retinal or choroidal disease, visual field loss attributable to an underlying condition (such as stroke, optic neuritis, trauma, etc.) with stable function for less than the 5 immediately preceding years, systemic medication known to affect the eye and/or the primary visual pathway, and an inability to achieve reliable ERG recordings and/or perimetry, as defined by the respective eligibility criteria.

The placebo arm comprised 47 participants at baseline. Nine participants withdrew during the course of the study, five because of adverse events and four for non-medical reasons; none withdrew due to ophthalmic events. Of the remaining 38 participants (mean [SD] age, 62.7 [8.1] years; median, 64.5 years; interquartile range, 13 years; range, 48–79 years), 21 were male and 17 female. Three centers contributed 7, 8, and 10 participants, respectively; two centers had 3 and 4 participants; and four centers each had between 1 and 2 participants.

Two of the remaining participants were current smokers, 17 had ceased smoking, and 19 had never smoked. Ten participants had diabetes mellitus. Twenty-one participants exhibited one or more ocular conditions that were within the inclusion criteria and typical for age. The most frequent of these conditions was age-related cataract (nine cases). A description of the type and extent of lenticular opacity at inclusion/baseline was not required in the study protocol approved by the EMA. However, cataract surgery within the time course of the study was an exclusion criterion, and each participant was requested not to elect for ophthalmic surgery unless as an emergency procedure. In the placebo group, an emergent cortical cataract was reported in one individual, and there were no cases of either cataract surgery or emergency ocular surgery. Cataract, in addition, would not be expected to affect the full-field ERG. The nine cataract cases and individuals with mild age-related macular degeneration (two cases) and retinal hemorrhage (two cases) were included at the discretion of the examining ophthalmologist.

The study was conducted in accordance with the ethical principles stated in the 1964 Declaration of Helsinki and as revised in Seoul 2008. All participants gave written informed consent prior to visual function testing.

### Visual Function Testing

BCVA was measured in each eye, separately, at 4 m using the Early Treatment Diabetic Retinopathy Study high-contrast chart (EVA-Tester; STZ Biomed, Tübingen, Germany) and converted into logMAR units.

CVis was measured in each eye, separately, using the Lanthony desaturated D15 color vision arrangement test (Luneau, Paris, France) displayed within a D65 standard illuminant test box (Judge QC; X-Rite, Inc., Grand Rapids, MI, USA) at a luminance of 270 lux white light. The total error score (TES) was calculated by evaluation software in the public domain.

ERG was performed for each eye, simultaneously, in compliance with the ISCEV Standard pertaining at the time of study initiation^25^ with single-use Dawson–Trick–Litzkow fiber electrodes and the Espion E2 device (Diagnosys LLC, Cambridge, UK). Custom software within the Espion device defaulted to a fixed 20-minute dark adapted (DA) and a fixed 10-minute light adapted (LA) period. Reference electrodes were placed on the zygomatic fossae and the ground electrode on the central forehead. Pupils were dilated with either 0.5% or 1% tropicamide, as appropriate. All participants achieved a dilated pupil diameter of at least 4 mm. Each set of electrodes had a unique serial number that was recorded in the participant's digital CRF file at each visit.

Each ERG stimulus was presented three times with the responses averaged to a “result”, including automatic and manual artifact rejection, thereby facilitating recordings with low noise. Each result was repeated at least twice at each session. The ERGs were initially evaluated by each center, in terms of cursor position, and then transferred to a central database to which the central reader had online access and analyzed using custom clinical trial software (Diagnosys LLC). The central reader could “replay” each session, see each individual recording, check the markers, compare with previous recordings, make comments, ask for repeat etc. (see Annex D of the Supplementary Material). For further details, see Zrenner.^26^ The central reader assessed data quality based on signal-to-noise ratio; consistency of stimulus and recording conditions; the absence of artifacts at critical phases of the response; the correct setting of the cursors for the a-wave trough and b-wave peak; and the completeness of the recordings in relation to each stimulus parameter of the protocol. Any change to the cursor position by the central reader was documented in an audit trail. If a response was technically unsatisfactory, the central reader requested an additional recording; however, the final decision was left to the ophthalmological investigator.

SKP was undertaken on each eye, followed, after a 5- to 10-minute rest period by SAP, using the Octopus 101 perimeter (Haag-Streit, Köniz, Switzerland).^30^ For each perimetric modality, the right eye was examined first. SKP was performed along each 15° meridian at an angular velocity of 3°/s, initially with the III4e stimulus and then with the I3e stimulus. The angular extent of each isopter was corrected by the perimeter software for the reaction time of the given participant determined from the standard presentation of three reaction time vectors placed within 30° eccentricity from fixation.^19^^,^^31^ The angular extent of the blind spot was determined from the presumed center along each 30° meridian with the I4e stimulus presented at 2°/s. The perimeter automatically calculated the area within each isopter. The reliability criteria comprised a reaction time of between 200 and 1500 ms in either eye and an areal extent of the blind spot of less than 43 square degrees.

SAP was undertaken with stimulus size III and Program G1, which estimates the threshold at each of 64 locations within 30° eccentricity from fixation, using a 4-2-1 dB staircase threshold assessment strategy, with the distance refraction and the near addition, appropriate for the viewing distance of the perimeter bowl, in situ. For each subsequent visit, the threshold derived at each individual location during the previous examination was used as the starting value of the staircase for the corresponding location to reduce the examination duration. The reliability criteria in either eye were ≤30% incorrect responses to the false-positive and false-negative catch trials, respectively. Fixation stability during each type of perimetry was continuously evaluated by the examiner via the perimeter monitor.

### Statistical Analysis

A descriptive analysis was undertaken for each eye separately on the absolute and proportionate change in the measured values between the baseline and the 36-month visit, that is, [month 36 (M36) – baseline (M0)] and [[month 36 (M36) – baseline (M0)] / baseline (M0) × 100], respectively, for the ERG amplitudes and peak times, the Lanthony desaturated total error score, each isopter area, and the Mean Defect (MD) visual field index.

The inferential analysis was undertaken using a two-tailed Wilcoxon signed-rank test without adjustment, based on the Hodges and Lehman estimator^32^ and using Walsh averages, to determine whether the difference in the estimated absolute values over the 36 months and also that of the estimated proportionate values was statistically significantly different from baseline. A non-parametric approach with the Hodges and Lehman estimator was adopted due to the relatively small size of the cohort. The Hodges and Lehman estimator was used to obtain a better point estimate (i.e., of the median difference). The Walsh averages procedure was used to obtain the two-sided 95% confidence interval (CI) associated with each point estimate. This is an evaluation procedure common in clinical trials.^33^ The means were estimated to facilitate a comparison with the estimated medians: an underlying assumption of the Hodges and Lehmann estimator is symmetry of the distribution around the median.

To mitigate against a Type I error arising from the multiple comparison of medians, a stringent rule was applied, based on three criteria, two of which had to be separately and also conditionally satisfied. First, any statistically significant result was required to be present in the fellow eye; second, if the absolute difference was significantly different from zero, then the proportionate difference also had to be statistically different from zero; finally, the significance levels were evaluated at *P* ≤ 0.025, *P* ≤ 0.01, and *P* ≤ 0.001; a value of *P* < 0.05 was considered only in the presence of other consistent statistically significant values at *P* ≤ 0.025 for the given visual function.

## Results

All 38 participants completed the BCVA, CVis, and ERG protocols; 36 completed the SKP and 32 the SAP protocols.

**Figure 1.**
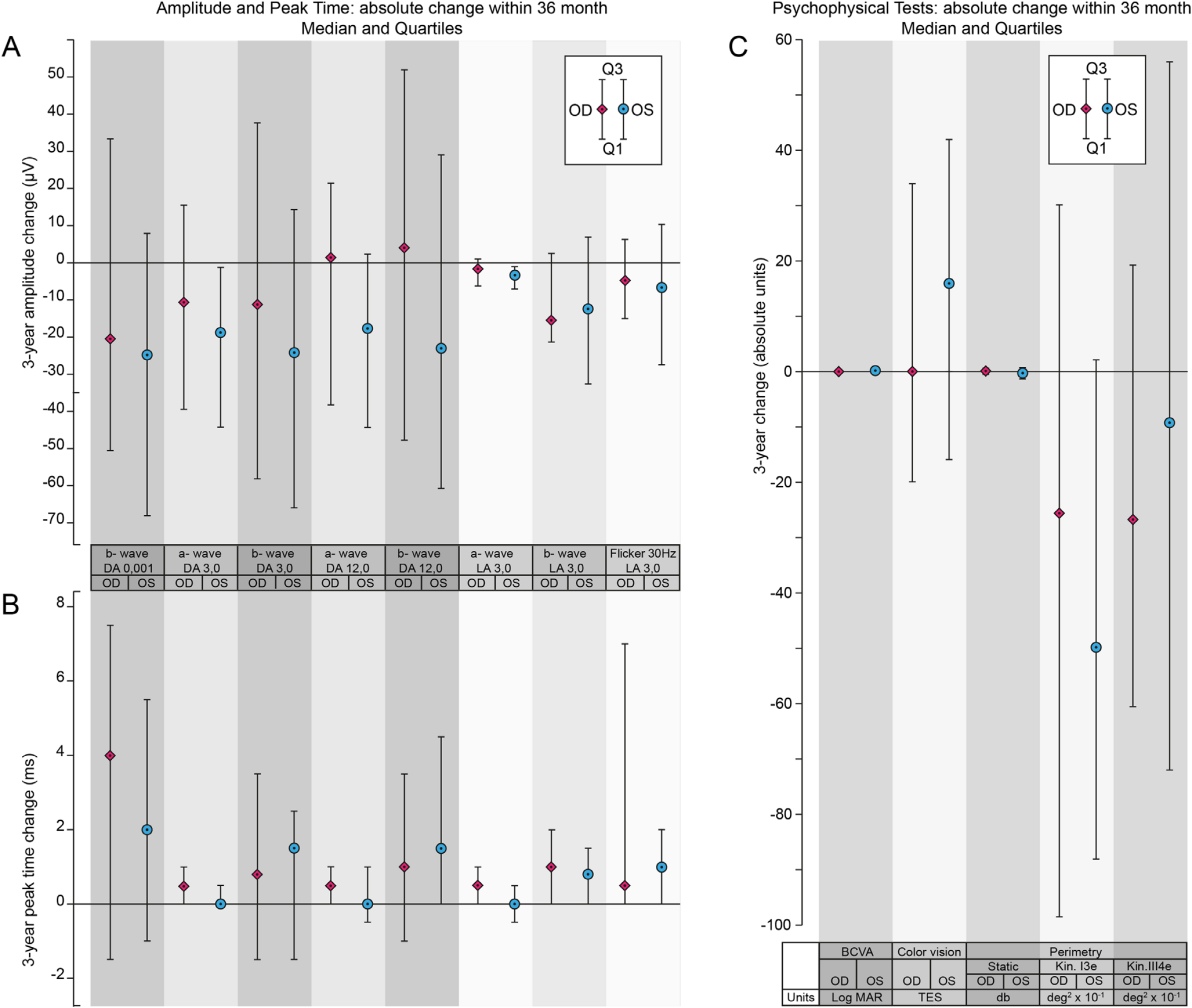
The median and interquartile range (Q1; Q3) of the absolute difference (OD, *diamonds*; OS, *circles*) in the measured values over the 36 months for the ERG amplitudes (**A**) and ERG peak times (**B**), in the DA and LA stage, and for the visual function tests (**C**).

**Figure 2.**
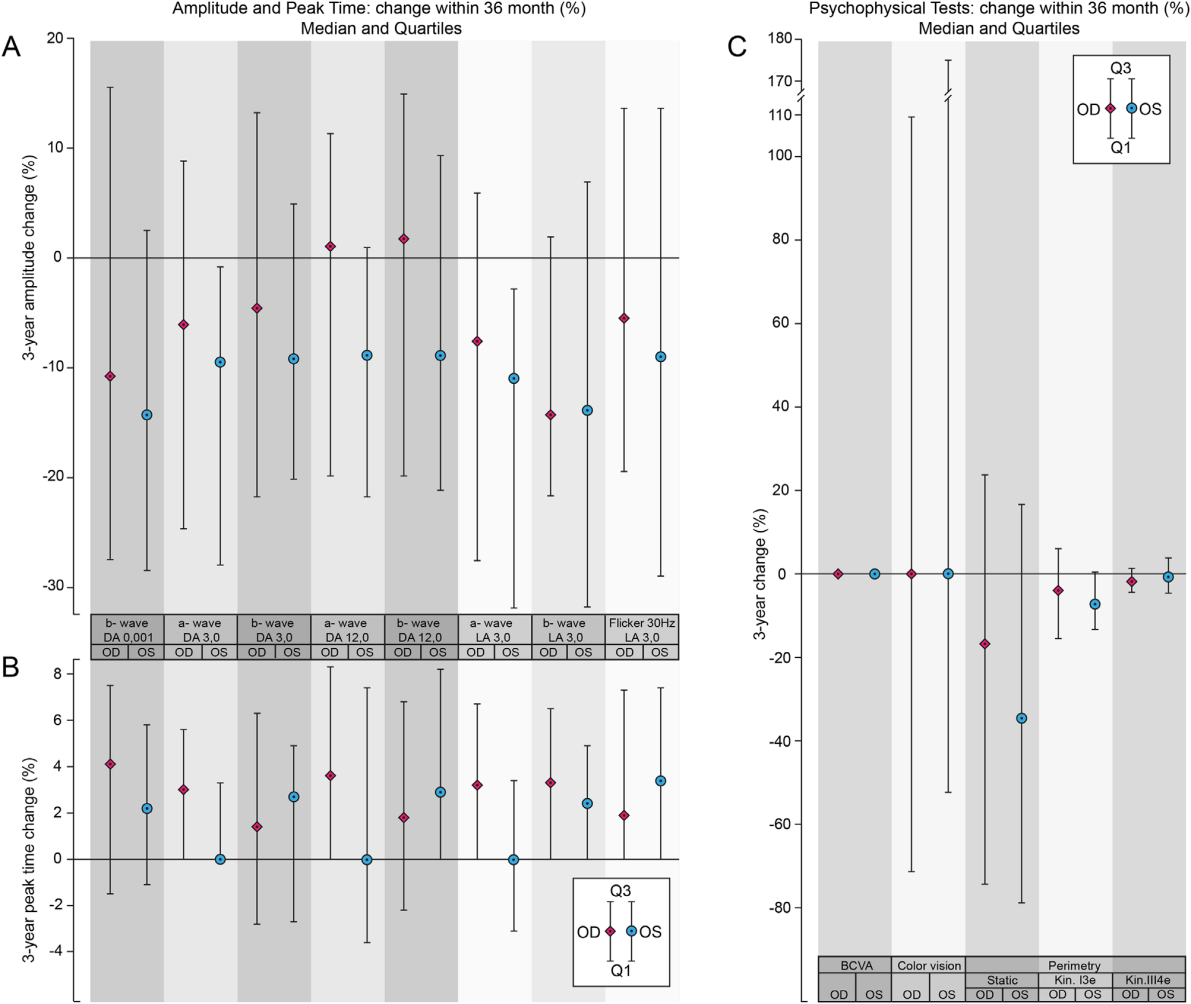
The median and interquartile range (Q1; Q3) of the proportionate difference (OD, *diamonds*; OS, *circles*) in the measured values over the 36 months for the ERG amplitudes (**A**) and ERG peak times (**B**), in the DA and LA stage, and for the visual function tests (**C**).

The descriptive statistics for the differences in the measured value for each eye over the 36 months are illustrated graphically (in absolute terms in Fig. 1 and in proportionate terms in Fig. 2) for the ERG amplitudes and peak times and for each of the various visual function tests. The measured values at baseline (designated as M0) and M36 together with the absolute and proportionate differences (M36 – M0) for all parameters are given in Annex B of the Supplemental Material, Table B1 for the right eye and Table B2 for the left eye; a deterioration in visual function is indicated by a negative value for all parameters with the exception of the ERG peak time and the TES of the Lanthony desaturated color vision test, in which a deterioration is indicated by a positive sign.

The estimated absolute and proportionate median differences over the 36 months (E) and the two-sided 95% CIs associated with the estimated median, as well as the significance levels arising from the Wilcoxon signed-rank test (*P* ≤ 0.05, *P* ≤ 0.025, *P* ≤ 0.01, and *P* ≤ 0.001), for the ERG amplitudes and peak times are given in Table 1 for each eye separately and those for BCVA, CVis, SAP and SKP in the Table 2, for each eye separately. A negative value indicates a deterioration in visual function for each parameter except those for the ERG peak time and for the TES of the Lanthony desaturated color vision test, in which a positive sign indicated a deterioration.

**Table 1.** Significance Levels Associated With the Estimated (E) Absolute and Proportionate Median Differences in the ERG-Amplitudes and Peak Times and in the ERG parameters for the Right (OD) and Left (OS) Eyes over the 36 Months

	Right Eye (OD)					Left Eye (OS)					Difference Between Eyes	
	OD	OD Absolute		OD Proportional (%)		OS	OS Absolute		OS Proportional (%)		(OD – OS) Absolute	(OD – OS) Proportional (%)
	*n*	M0 – M36		(M36 – M0)/M0 × 100		*n*	M0 – M36		(M36 – M0)/M0 × 100		Delta (OD – OS)	Delta (OD – OS)
1	2	3	4	5	6	7	8	9	10	11	12	13
Two-Sided *P* Value of the Paired Wilcoxon Signed-Rank Test		*P*	E (95% CI)	*P*	E (95% CI)		*P*	E (95% CI)	*P*	E (95% CI)	Mean (SD)	Mean (SD)
*DA 0.01 b-wave* *amplitude (µV)*	38	0.137	−14.1 (−36.85 to 5.35)	0.183	−7.25 (−17.55 to 2.75)	38	***0.019***	−25.8 (−49.45 to −3.95)	*0.034*	−*12.25*(−21.4 to −0.95)	6 .0 (65.3)	−0.1 (29.8)
***DA 3.0 a-wave*** ***amplitude (µV)***	38	*0.04*	*14.35* (0.40 to 28.85)	*0.042*	−7.9 (−16.4 to −0.05)	38	**0.003**	20.25 (6.85 to 34.40)	**0.005**	−**11** (−18.2 to −3.6)	3.4 (63.1)	−1.0 (30.2)
*DA 3.0 b-wave* *amplitude (µV)*	38	0.254	−13.65 (39.35 to 10.70)	0.201	−5.2 (−13.7 to 3.65)	38	***0.024***	−24.0 (−47.6 to −3.0)	***0.018***	−*8.2* (−15.25 to −1.0)	2.4 (93.5)	0.3 (29.4)
*DA 12.0 a-wave* *amplitude (µV)*	38	0.554	6.95 (−11.05 to 25.95)	0.608	−2.35 (−11.45 to 5.65)	38	**0.01**	20.35 (5.85 to 35.35)	***0.021***	−*9.25* (−15.7 to −2.2)	−5.1 (66.6)	4.0 (26.5)
*DA 12.0 b-wave* *amplitude (µV)*	38	0.848	−3.3 (−33.45 to 23.3)	0.727	−1.6 (−11.25 to 8.05)	38	0.067	−21.5 (−43.7 to 1.3)	0.086	−7.15 (−13.7 to 0.95)	11.8 (92)	4.0 (28)
*LA 3.0 a-wave* *amplitude (µV)*	37	0.088	2.0 (−0.4 to 0.5)	0.102	−7.6 (−16.5 to 1.85)	38	**0.003**	3.7 (1.6 to 6.25)	**0.004**	−13.2 (−22.4 to −5.15)	−1.0 (6.9)	4.6 (27.1)
***LA 3.0 b-wave*** ***amplitude (µV)***	37	**0.005**	−12.1 (−19.9 to −4.1)	**0.003**	−11.56 (−18.3 to −3.85)	38	**0.004**	−14.6 (−25.8 to −4.6)	**0.003**	−13.2 (−20.7 to −4.05)	3.1 (24.1)	1.5 (22.3)
*LA 3.0 30-Hz flicker* *amplitude* *(µV)*	37	0.122	−5.25 (−12.15 to 1.6)	0.152	−5.5 (−13.15 to 2.9)	37	***0.015***	−9.4 (−17.5 to −2.35)	0.068	−8.15 (−17.75 to 1.1)	4.3 (22.2)	2.7 (24.5)
***DA 0.01 b-wave*** ***peak time (ms)***	38	**0.005**	3.25 (1.25 to 5.7)	**0.003**	3.65 (1.3 to 6.1)	38	*0.028*	2.25 (0.25 to 4.5)	***0.025***	*2.4* (0.25 to 4.6)	1.6 (8.1)	1.7 (8.1)
***DA 3.0 a-wave*** ***peak time (ms)***	38	**≤0.001**	0.5 (0.25 to 0.75)	**0.001**	2.8 (1.45 to 4.35)	38	***0.024***	0.25 (0.0 to 0.5)	**0.006**	1.55 (0.0 to 3.1)	0.1 (0.8)	0.8 (4.7)
*DA 3.0 b-wave* *peak time (ms)*	38	0.21	0.75 (−05 to 2.0)	0.213	1.5 (−0.85 to 3.85)	38	0.056	0.95 (0.0 to 1.75)	0.052	1.65 (0.0 to 3.45)	−0.5 (4.0)	−0.8 (7.3)
*DA 12.0 a-wave* *peak time (ms)*	38	**0.008**	0.5 (0.2 to 0.75)	**0.007**	3.65 (1.55 to 6.10)	38	0.231	0.25 (−0.25 to 0.5)	0.142	1.8 (−1.55 to 4.0)	0.2 (0.9)	1.7 (7.4)
*DA 12.0 b-wave* *peak time (ms)*	38	0.16	0.75 (−0.5 to 2.25)	0.161	1.6 (−0.6 to 4.4)	38	*0.028*	1.5 (0.25 to 2.8)	***0.017***	*3.15* (0.65 to 5.8)	−0.8 (6.9)	−1.7 (14.1)
*LA 3.0 a-wave* *peak time (ms)*	37	*0.043*	0.5 (0.0 to 0.75)	***0.021***	3.2 (0.15 to 5.05)	38	0.289	0.25 (0.0 to 0.5)	0.241	1.55 (−0.05 to 3.3)	0.1 (1.3)	0.8 (9.2)
***LA 3.0 b-wave*** ***peak time (ms)***	37	**≤0.0001**	1.0 (0.5 to 1.5)	**≤0.0001**	3.3 (1.65 to 4.85)	38	**≤0.001**	0.75 (0.25 to 1.25)	**≤0.001**	2.45 (0.9 to 4.0)	0.1 (0.6)	0.4 (2.1)
***LA 3.0 30-Hz* *flicker*** ***peak time (ms)***	37	**≤0.0001**	1.0 (0.5 to 1.5)	**≤0.0001**	3.5 (1.8 to 5.3)	37	**≤0.001**	1.0 (0.5 to 1.25)	**≤0.001**	3.2 (1.65 to 4.9)	0.1 (1.0)	0.5 (3.8)

*P* ≤ 0.05 (italics, underlined), *P* ≤ 0.025 (italics, bold), *P* ≤ 0.01 (straight bold), and *P* ≤ 0.001 (bold, underlined). CI represents the 95% confidence interval of the corresponding estimated median difference E. A positive absolute change (M36 – M0) in the amplitude for an a-wave indicates a less negative amplitude (i.e., a reduction in amplitude, as expressed by the negative proportionate difference).

**Table 2.** Significance Levels Associated With the Estimated (E) Absolute and Proportionate Median Differences in BCVA, Color Vision and Visual Field for the Right (OD) and Left (OS) Eyes over the 36 Months

	Right Eye (OD)					Left Eye (OS)					Difference Between Eyes	
	OD	OD Absolute		OD Proportional (%)		OS	OS Absolute		OS Proportional (%)		(OD – OS) Absolute	(OD – OS) Proportional (%)
	*n*	M0 – M36		(M36 – M0)/M0 × 100		*n*	M0 – M36		(M36 – M0)/M0 × 100		Delta (OD – OS)	Delta (OD – OS)
1	2	3	4	5	6	7	8	9	10	11	12	13
Two-Sided *P* Value of the Paired Wilcoxon Signed-Rank Test		*P*	E (95% CI)	*P*	E (95% CI)		*P*	E (95% CI)	*P*	E (95% CI)	Mean (SD)	Mean (SD)
*BCVA (logMAR)*	38	1	0.0 (0–0 to 0.0)	—	—	38	0.404	0.0 (0.00 to 0.05)	—	—	0.0 (0.1)	−12.5 (67.8)
*Color vision (TES)*	38	0.299	7.0 (−8.0 to 24.0)	0.431	16.0 (−33.5 to 75.0)	38	0.158	14.0 (−6.0 to 33.0)	0.225	43.0 (−23 to 114)	−3.4 (54.9)	−47 (227.3)
*VF* *static (MD,* *db)*	35	0.973	0.0 db (−0.45 to 0.45)	0.248	−15.9 (−53.5 to 14.35)	33	0.316	−0.25 db (−0.8 to 0.3)	*0.033*	*−33.95*(−63.05 to −2.35)	0.2 (1.0)	15.7 (152.9)
***VF*** ***k*** ***inetic I3e* *(deg^2^)***	36	*0.036*	−317.75 (−719.5 to −9.0)	*0.045*	−4.95 (−11.4 to 0.00)	37	**0.001**	−451.5 (719.5 to −206)	**0.001**	−7.15 (−11.55 to −3)	72.8 (1032)	0.8 (16.9)
*VF* *kinetic III4e (deg^2^)*	36	0.096	−207.5 (−430 to 48)	0.106	−1.6 (−3.2 to 0.55)	37	0.548	−80.0 (−415.5 to 258)	0.563	−0.55 (−3.05 to 1.9)	107.3 (1046)	−0.9 (9.4)

*P* ≤ 0.05 (italics, underlined), *P* ≤ 0.025 (italics, bold), *P* ≤ 0.01 (straight bold), and *P* ≤ 0.001 (bold, underlined). CI represents the 95% confidence interval of the corresponding estimated median difference E.

Statistically significant deteriorations both in absolute and in proportionate terms were present for the estimated values of the ERG DA 3.0 a-wave amplitude, the LA 3.0 b-wave amplitude, the DA 0.01 b-wave peak time, the DA 3.0 a-wave peak time, the LA 3.0 b-wave peak time, the LA 3.0 30-Hz flicker peak time, and the I3e isopter area.

The differences in the estimated absolute values of the BCVA, the TES for the Lanthony saturated D15 color vision, the MD for SAP, and the III4e isopter area of SKP did not reach statistical significance in either eye.

The interocular differences (Table 1 and Table 2, columns 12 and 13) in the estimated absolute and proportionate mean differences over the 36 months were not significant for any of the visual function parameters. However, five of the ERG parameters showed a statistically significant absolute and proportionate difference for the left eye only. The reason for this is unclear given that the ERG for the right and left eyes is recorded simultaneously.

Examples of differences over the 36 months for each participant by age at enrollment are presented graphically in Annex C in Figure S1 of the Supplementary Material for the LA 3.0 ERG b-wave amplitude (A) and peak time (B), the proportionate difference in the LA 3.0 30-Hz flicker ERG (C), the Mean Defect visual field index (D), and the I3e (E) and the III4e isopter (F) areas. The magnitude of the deterioration appeared to be independent of age at enrollment.

## Discussion

Statistically significant reductions in visual function, primarily that of the estimated ERG but also that of the I3e isopter area, were detected in a cohort of participants with chronic stable angina pectoris (mean age at baseline of 62.7 years) and clinically normal visual function, over a 36-month follow-up period. These findings are remarkable for three reasons. First, a deterioration in visual function was detectable over an interval as short as 36 months. Second, the deterioration was detected in a multicenter study involving 11 ophthalmology centers, thereby attesting to the high quality of the data acquisition. Third, the deterioration occurred in both objective (the ERG) and subjective (the I3e isopter of SKP) visual function tests. An immediately obvious explanation for such deteriorations in performance is the normal physiologic decline with increasing age. However, given the specific characteristics of the cohort, including chronic stable angina pectoris, other covarying causes cannot be completely ruled out, although chronic stable angina pectoris, per se, is not known to affect visual function.

Essentially, all structures in the eye are prone to physiologic aging. The associated deterioration in visual function can also be accompanied by additional dysfunction arising from concomitant morbidities. Thus, the visual function characteristics of any control group are determined by a wide spectrum of possible etiologies, the prevalence and/or impact of which increases with age and/or with the worsening of the comorbidity. In safety trials, the representative nature of the control and treatment groups to that of their respective populations is a key factor. In the current study, the underlying characteristic of the control group was angina pectoris. However, there was no suggestion from the various tests that the ocular health of the participants differed from that of those without stable chronic angina pectoris in age-matched control groups from other studies. There is no literature concerned with visual function in angina pectoris other than our previous publication.^26^

The prevalence of diabetic retinopathy is greater among patients with cardiovascular diseases than those without.^34^ Vascular eye disease was an exclusion criterion and no such cases emerged during the study. However, subclinical microvascular changes occurring during the study cannot be ruled out. Renal and severe hepatic diseases and concomitant medication with possible effects on the visual system were further exclusion factors (see Methods and Annex A of the Supplementary Material).

The distributions of the various visual function outcomes in the control group were each similar to those from other studies in which the deterioration in visual function is more clearly attributable to physiologic aging. For example, the mean and 95th percentile of the TES for the cohort as a whole, both at M0 (20; 120) and at M36 (30; 194), compare favorably with those from individuals aged 60 to 69 years (12; 170) and aged 70 to 79 years (150; 195)^35^, suggesting that the clarity of the crystalline lens in the current study was no different from that of the comparison study. The change in the distribution of the MD index, which is an index corrected for physiologic aging, over the 3 years was not significantly different from zero and further supports the assumption that chronic stable angina pectoris did not substantially influence the results, at least for SAP. It is reasonable to assume that the physiologic aging process was the predominant factor underpinning the distributions of changes in the current study. If a control group is absent in studies that resemble the current study, the data reported here may help to separate treatment effects from aging effects.

The estimated BCVA did not appear to deteriorate over the 36 months of the study. Such a finding is compatible with a decline of one letter per year in those aged between 65 and 84 years old when followed up after 2-, 6-, and 8-year periods.^36^

The estimated Mean Defect visual field index, averaged across the two eyes, did not change over the 36 months. Such a finding was not unexpected given that the Mean Defect is the mean of the differences, at each stimulus location, between the “cross-sectional” age-corrected normal value, derived “cross-sectionally,” and the measured value.

The reduction in the I3e isopter area over the 36 months compared with the stability of the III4e isopter area is consistent with the cross-sectional evidence.^19^ However, the reduction in area over an interval as short as 36 months is a novel observation. It should be noted that the III4e stimulus is associated with a ceiling effect (i.e., the III4e isopter in the temporal field extends beyond the available surface of the perimeter bowl).

The increase in the estimated median difference in the ERG peak time of the DA bright-flash a-wave after 36 months, although statistically significant, was less than 0.5 ms (Table 1). Such a small change is not clinically important; however, the magnitude of the difference confirms the known robust nature of the a-wave peak time.^11^ Nevertheless, such a small deterioration potentially attributable to aging has not previously been reported over a period of 36 months.

The plots of the deteriorations over the 36 months, by age of the participant at enrollment, of the various parameters that were statistically significant at *P* ≤ 0.025 (some of which are illustrated in Fig. S1 of Annex G) convincingly show the reduction in ERG amplitudes, the increase in ERG peak times, and the reduction in the I3e isopter area. Within the limits of the data set, the magnitudes of the differences appeared to be independent of age. However, the identification of any possible age dependency in the “longitudinal” deterioration in the MD over the 36 months was prevented by the wide dispersion around the no-difference (i.e., zero) line present at approximately 67 to 70 years of age (Fig. S1.D of Annex G). The increasing dispersion with age covaried, in part, with the heterogeneity in the magnitude of the MD index within- and between-participants and with the perimetric fatigue effect,^37^ both of which are more likely to occur with increasing age. The within- and between-test variability increases with a reduction in sensitivity reaching a maximum between 18 and 12 dB, after which it declines.^38^^–^^41^ The perimetric fatigue effect for SAP^37^ describes the reduction in the measured sensitivity (and, therefore, an associated increase in the variability), which worsens during the course of the visual field examination within each eye and between the first and second eye examined at any given visit. A fatigue effect in SKP, attributed to sequential presentations of the stimulus, manifests as a reduction in the III4e isopter.^42^ The field of the right eye in the present study was always examined before that of the left eye for both SAP and SKP.

The compatibility of the “longitudinal” deteriorations in visual function present in the current study with those in the literature determined “cross-sectionally” supports the assumption that the changes were due to aging rather than to the underlying chronic angina pectoris. The magnitude of the deterioration over the 36 months in the ERG and in the I3e isopter is sufficiently large to consider the use of a correction to separate the deterioration associated with either aging and/or the disease from the treatment-related change in endpoint values in safety and/or efficiency trials, as well as in natural history studies, of a similar or longer duration to the current study. Such an approach would facilitate correct apportion either of apparent adverse events or of beneficial outcomes, attributable to the interventional effect and would be appropriate for trials of pharmacologic or gene therapy-related interventions for slowly progressive eye disease. The magnitude of the deteriorations is likely to be of greater concern in clinical trials than in clinical practice.

The visual functions in the current study are mediated by a variety of ocular structures. The Ganzfeld-ERG is a global electrical retinal response consisting of individual components derived from different retinal layers; the a-wave depends strongly on the integrity of the photoreceptor outer segments and the b-wave on the integrity of the inner nuclear layer, principally the bipolar cells. High-contrast BCVA, color vision, and perimetry each involve the integrity of the photoreceptors, the postsynaptic retinal neurons, and the ascending pathways and cortical structures, in a chain-like manner, including regional differentiation.

The structure of the normal retina, revealed by optical coherence tomography (OCT), exhibits age-related changes. The thickness of the ganglion cell layer, the inner plexiform layer, the inner nuclear layer, and the outer nuclear layer, including the Henle fibers, each declines at approximately 0.07 µm/y when determined cross-sectionally.^23^ The rate of decline in the thickness of the outer and inner segments of the photoreceptors is much slower while that of the retinal nerve fiber layer, the outer plexiform layer, and the retinal pigment epithelium layer exhibits little change with age.^23^ Other estimates of the age-related reduction in the retinal nerve fiber layer of the normal eye vary, depending on the quadrant, from <1.8 µm/y (95% CI) over a 30-month longitudinal study^43^ to 0.44 µm/y over a 2- to 6-year longitudinal study^44^ or to a reduction of 2.1% per decade.^22^ However, the reduction in the retinal nerve fiber layer and the inner plexiform layer thicknesses with age may be greater at the macula.^45^

The proportionate changes over the 36 months in the a-wave amplitudes and peak times are less than those of the b-wave under both dark- and light-adaptation. These differences are compatible with the slight differences in the rate of thinning with age between the outer and inner retinal layers, respectively.^23^ However, that significant alterations occurred in both the rod and cone-system–derived ERG parameters over the 36 months, despite the small morphologic changes in these layers by OCT, is further evidence to suggest that total reliance on structural rather than functional measures in clinical trials, in relation to the efficacy or to the safety of a novel intervention, may be inappropriate. The rod ERG a-wave amplitude exhibits a 0.01 log unit reduction per decade,^46^ which compares favorably to that of the rod system sensitivity of 0.06 log unit per decade. The additional reduction in the cone ERGs in the present study implies that the ERG, under strictly controlled conditions, may be more sensitive than the current technology for OCT. The increase in peak time and the reduction in amplitude with increasing age for both the dark- and the light-adapted ERGs are established for ophthalmologically normal individuals,^8^^–^^11^ although the magnitudes vary between laboratories. The maximum amplitudes in the rod and cone responses at 70 years of age are approximately half that of those between 15 and 24 years,^9^ and the amplitudes in a septuagenarian age group are 25% to 40% lower than in those between 20 and 50 years of age.^11^

It is reasonable to assume that the physiologic aging process was the predominant factor underpinning the distributions of change over the 3 years in the current study. This suggests the need for experimental designs that incorporate an age-corrected control group that is appropriate for the specific study. This may be especially important in a phase IV clinical trial without a control group. The novel finding of statistically significant changes over 36 months in this longitudinal multicenter trial also underlines the importance of a robust methodology incorporating identical and standardized techniques, central reader evaluation, and stringent quality control of the data acquisition.

## Conclusion

Potential age-related absolute changes in high-contrast BCVA, color vision, electroretinography, SKP, and SAP were assessed over 36 months in the group randomized to placebo of a well-controlled highly standardized double-blind multicenter trial involving participants with chronic stable angina pectoris and an acceptable standard of ocular health for their age range (48–79 years). Statistically significant deteriorations in each eye were present for several ERG parameters and for the I3e isopter. High-contrast BCVA and color vision did not change over the 36 months. A correction factor for these changes could be useful in future trials (for details, see Annex B in the Supplementary Material).

## Supplementary Material

Supplement 1
