# Supplementary material for: Three-Year Changes in Visual Function in the Placebo Group of a Randomized Double-Blind International Multicenter Safety Study: Analysis of Electroretinography, Perimetry, Color Vision, and Visual Acuity in Individuals With Chronic Stable Angina Pectoris
Source: Transl Vis Sci Technol. 2022 Jan 4;11(1):2. doi: 10.1167/tvst.11.1.2 (PMC8742521; doi:10.1167/tvst.11.1.2)
Supplement: Supplement 1 [file tvst-11-1-2_s001.pdf]

SUPPLEMENTAL MATERIAL

TVST Article:

Three-Year Changes in Visual Function in the Placebo Group of a Randomized Double-Blind International Multicenter Safety Study: Analysis of Electro-retinography, Perimetry, Color Vision, and Visual Acuity in Individuals with Chronic Stable Angina Pectoris

Eberhart Zrenner[1,2], Graham E. Holder[3–5], Ulrich Schiefer[1,6], and John M. Wild[7]

1 Center for Ophthalmology, University of Tübingen, Tübingen, Germany
2 Werner Reichardt Center for Integrative Neuroscience (CIN), University of Tübingen, Tübingen, Germany
3 Moorfields Eye Hospital, London, UK
4 University College London, Institute of Ophthalmology, London, UK
5 Department of Ophthalmology, Yong Loo Lin School of Medicine, National University of Singapore, Singapore
6 Competence Center Vision Research, University of Applied Sciences Aalen, Aalen, Germany
7 College of Biomedical Sciences, Cardiff University, Cardiff, UK

ANNEX A:

<u>Complete description of exclusion criteria:</u>

Cardiac exclusion criteria comprised clinically significant heart disease other than coronary artery disease; severe hypotension (systolic <90mmHg and diastolic <50mmHg); uncontrolled hypertension at rest (systolic >180mmHg or diastolic <100mmHg); acute myocardial infarction within 6 months of pre-selection; coronary bypass surgery within two months of pre-selection; coronary angioplasty within 6 months of pre-selection; high-grade coronary artery disease which had not been surgically by-passed or mechanically improved; and fibrillation, flutter and either pace-maker or cardioverter-defibrillator implantation.

Systemic exclusion criteria: the presence of hepatitis B surface antigens, and/or human immunodeficiency virus antibodies and/ or hepatitis C antibodies; history of severe psychiatric or behavioral disorder likely, in the investigator's judgement, to interfere with the study; history of serious abnormal drug reaction; existing, or potential, treatment with macrolide antibiotics (e.g. clarithromycin, erythromycin, josamycin, telithromycin), HIV inhibitors (e.g., nelfinavir and ritonavir), nefazodone, antiretroviral drugs or azoles and antifungals, e.g. ketoconazole; treatment with treatment with unauthorized concomitant medication (see below) that could not be interrupted for the duration of the study; treatment with bepridil or amiodarone within 7 days and 3 months, respectively, prior to pre-selection; contra-indication to ivabradine including 3rd degree atrio-ventricular block (AVBIII), resting bradycardia (heart rate < 50 bpm) or sick sinus syndrome; and lactose intolerance.

ANNEX B

The summary statistics for the measured values for the ERG amplitudes (A) and peak times (B) and the BCVA, CVis and VF (C), at baseline (M0) and 36 months (M36) (columns 3 and 4 respectively) and for the absolute and proportionate differences between M0 and M36 (columns 5 and 6 respectively). Note for the

negative a-wave amplitudes: a reduction in amplitude over the 36 months results in a positive absolute value (meaning “less negative”) and a negative proportionate value (indicating loss of amplitude). Table B1 shows the data for the right eye, Table B2 for the left eye.

TABLE B1: Descriptive statistics for the baseline and M36 outcomes and for the differences over the 36 months: RIGHT EYE

		Table B1: Right eye (OD)			
Parameters	STAT	Baseline (M0)	M36A under treatment	M36A under treatment-Baseline (M0)	M36A under treatment-Baseline (M0)/Baseline (M0) x 100 $
DA 0.01 b-wave Amplitude (µV)	N	38	38	38	38
	Median	196.0	182.1	-20.6	-10.8
	Min ; Max	100.9 ; 393.6	52.1 ; 354.2	-197.7 ; 134.1	-70.1 ; 96.6
	P5;P95	118.4;345.5	62.9;327.2	-167.4;62.3	-57.5;43.0
	Q1 ; Q3	158.1 ; 272.6	131.1 ; 262.6	-50.9 ; 33.4	-27.5 ; 15.6
	Mean ± SD	210.4 ± 70.9	191.7 ± 79.7	-18.7 ± 66.7	-7.0 ± 32.4
	SEM	11.5	12.9	10.8	5.3
DA 3.0 a-wave Amplitude (µV)	N	38	38	38	38
	Median	-169.8	-157.4	10.6	-6.1
	Min ; Max	-349.7 ; -90.7	-307.9 ; -51.2	-84.0 ; 181.3	-58.5 ; 48.6
	P5;P95	-337.1;-105.4	-298.7;-86.2	-50.3;145.1	-51.8;28.2
	Q1 ; Q3	-192.4 ; -144.3	-179.1 ; -117.3	-15.6 ; 39.5	-24.7 ; 8.9
	Mean ± SD	-179.3 ± 57.4	-161.1 ± 56.7	18.2 ± 50.5	-8.4 ± 23.9
	SEM	9.3	9.2	8.2	3.9
DA 3.0 b-wave Amplitude (µV)	N	38	38	38	38
	Median	284.4	263.1	-11.3	-4.6
	Min ; Max	135.4 ; 488.4	77.2 ; 460.1	-229.0 ; 99.3	-53.3 ; 36.9
	P5;P95	163.0;462.8	102.0;448.8	-196.5;89.1	-48.6;33.7
	Q1 ; Q3	234.5 ; 349.5	206.9 ; 343.2	-58.2 ; 37.7	-21.8 ; 13.3
	Mean ± SD	295.1 ± 82.2	275.9 ± 95.8	-19.3 ± 77.5	-5.9 ± 24.2
	SEM	13.3	15.5	12.6	3.9
DA 12.0 a-wave Amplitude (µV)	N	38	38	38	38
	Median	-195.1	-199.5	-1.5	1.1
	Min ; Max	-452.3 ; -116.1	-369.9 ; -88.2	-119.1 ; 229.6	-53.1 ; 56.4
	P5;P95	-380.0;-126.5	-346.6;-89.4	-91.6;162.2	-50.8;48.0
	Q1 ; Q3	-228.8 ; -174.5	-235.5 ; -154.8	-21.5 ; 38.3	-19.9 ; 11.4
	Mean ± SD	-214.0 ± 70.1	-201.9 ± 67.4	12.1 ± 66.1	-3.1 ± 25.6
	SEM	11.4	10.9	10.7	4.1
DA 12.0 b-wave Amplitude (µV)	N	38	38	38	38
	Median	297.2	284.2	4.1	1.8
	Min ; Max	125.9 ; 551.0	77.3 ; 502.8	-279.4 ; 184.9	-50.7 ; 67.6
	P5;P95	144.0;505.4	113.5;499.7	-166.7;141.5	-50.4;39.5
	Q1 ; Q3	233.1 ; 359.8	231.4 ; 368.0	-47.8 ; 52.0	-19.9 ; 15.0
	Mean ± SD	306.3 ± 90.8	297.5 ± 106.1	-8.8 ± 92.8	-1.6 ± 27.0
	SEM	14.7	17.2	15.0	4.4
LA 3.0 a-wave Amplitude (µV)	N	37	37	37	37
	Median	-27.2	-23.7	1.6	-7.6
	Min ; Max	-50.3 ; -12.3	-43.2 ; -8.9	-10.9 ; 25.6	-57.7 ; 60.2
	P5;P95	-47.9;-16.4	-42.6;-11.9	-10.3;24.3	-54.4;47.2
	Q1 ; Q3	-31.5 ; -22.3	-31.2 ; -19.4	-1.1 ; 6.3	-27.6 ; 6.0
	Mean ± SD	-28.1 ± 9.1	-25.2 ± 8.6	3.0 ± 9.0	-7.1 ± 28.2
	SEM	1.5	1.4	1.5	4.6
LA 3.0 b-wave Amplitude (µV)	N	37	37	37	37
	Median	102.5	95.3	-15.5	-14.3
	Min ; Max	41.4 ; 174.7	25.4 ; 182.5	-67.7 ; 39.9	-51.0 ; 46.7
	P5;P95	50.2;166.1	28.8;162.0	-63.3;32.3	-44.0;38.5
	Q1 ; Q3	90.9 ; 132.3	80.0 ; 117.6	-21.4 ; 2.6	-21.7 ; 2.0
	Mean ± SD	110.9 ± 32.5	98.4 ± 34.2	-12.5 ± 25.7	-10.6 ± 22.8
	SEM	5.3	5.6	4.2	3.7

		Table B1: Right eye (OD)			
Parameters	STAT	Baseline (M0)	M36A under treatment	M36A under treatment-Baseline (M0)	M36A under treatment-Baseline (M0)/Baseline (M0) x 100 $
LA 3.0 30 Hz flick. Amplitude (µV)	N	37	37	37	37
	Median	87.8	78.3	-4.8	-5.5
	Min ; Max	19.3 ; 146.3	19.7 ; 177.6	-55.5 ; 37.4	-47.2 ; 58.4
	P5;P95	32.1;140.2	21.5;127.4	-48.2;35.8	-44.2;53.0
	Q1 ; Q3	66.6 ; 103.2	67.3 ; 97.8	-15.1 ; 6.4	-19.5 ; 6.2
	Mean ± SD	87.6 ± 28.9	81.8 ± 29.9	-5.8 ± 21.7	-4.6 ± 25.0
	SEM	4.7	4.9	3.6	4.1
DA 0.01 b-wave Peak time (ms)	N	38	38	38	38
	Median	96.5	100.0	4.0	4.1
	Min ; Max	83.0 ; 118.0	77.5 ; 144.5	-19.0 ; 39.5	-19.7 ; 41.9
	P5;P95	83.0;112.0	84.0;132.0	-12.0;39.5	-11.5;37.6
	Q1 ; Q3	91.5 ; 104.0	94.5 ; 106.0	-1.5 ; 7.5	-1.5 ; 7.9
	Mean ± SD	97.5 ± 8.1	101.8 ± 12.2	4.2 ± 10.6	4.5 ± 10.9
	SEM	1.3	2.0	1.7	1.8
DA 3.0 a-wave Peak time (ms)	N	38	38	38	38
	Median	16.5	17.0	0.5	3.0
	Min ; Max	14.5 ; 18.5	15.0 ; 19.0	-1.5 ; 2.5	-8.8 ; 16.1
	P5;P95	15.0;18.0	15.5;19.0	-1.0;2.0	-5.6;12.5
	Q1 ; Q3	16.0 ; 17.5	16.5 ; 18.0	0.0 ; 1.0	0.0 ; 5.6
	Mean ± SD	16.5 ± 1.0	17.0 ± 1.0	0.4 ± 0.8	2.8 ± 4.9
	SEM	0.2	0.2	0.1	0.8
DA 3.0 b-wave Peak time (ms)	N	38	38	38	38
	Median	53.5	53.8	0.8	1.4
	Min ; Max	44.0 ; 58.5	37.5 ; 60.0	-20.0 ; 8.7	-34.8 ; 19.8
	P5;P95	48.0;58.0	47.0;60.0	-6.5;7.0	-12.1;14.1
	Q1 ; Q3	52.0 ; 55.5	52.0 ; 56.5	-1.5 ; 3.5	-2.8 ; 6.3
	Mean ± SD	53.3 ± 2.9	53.7 ± 4.4	0.4 ± 4.8	1.0 ± 8.9
	SEM	0.5	0.7	0.8	1.4
DA 12.0 a-wave Peak time (ms)	N	38	38	38	38
	Median	13.0	13.5	0.5	3.6
	Min ; Max	10.5 ; 15.0	11.0 ; 16.5	-2.5 ; 2.5	-18.5 ; 22.7
	P5;P95	11.0;15.0	11.5;15.5	-2.5;2.5	-16.7;20.8
	Q1 ; Q3	12.0 ; 14.0	12.5 ; 14.5	0.0 ; 1.0	0.0 ; 8.3
	Mean ± SD	13.1 ± 1.2	13.5 ± 1.4	0.4 ± 1.1	3.6 ± 8.6
	SEM	0.2	0.2	0.2	1.4
DA 12.0 b-wave Peak time (ms)	N	38	38	38	38
	Median	51.5	52.0	1.0	1.8
	Min ; Max	40.5 ; 60.0	24.5 ; 63.0	-29.5 ; 16.5	-54.6 ; 36.3
	P5;P95	41.5;57.0	44.5;62.0	-5.0;13.5	-9.1;33.3
	Q1 ; Q3	50.0 ; 54.0	50.5 ; 55.0	-1.0 ; 3.5	-2.2 ; 6.8
	Mean ± SD	51.3 ± 4.2	52.2 ± 6.2	0.8 ± 6.9	2.2 ± 14.0
	SEM	0.7	1.0	1.1	2.3
LA 3.0 a-wave Peak time (ms)	N	37	37	37	37
	Median	15.0	15.5	0.5	3.2
	Min ; Max	12.0 ; 17.0	12.5 ; 17.5	-3.0 ; 2.5	-19.4 ; 17.9
	P5;P95	14.0;16.5	13.5;16.5	-2.5;2.0	-15.6;16.7
	Q1 ; Q3	14.5 ; 15.5	15.0 ; 16.0	0.0 ; 1.0	0.0 ; 6.7
	Mean ± SD	15.0 ± 0.9	15.3 ± 1.0	0.3 ± 1.2	2.2 ± 7.9
	SEM	0.1	0.2	0.2	1.3
LA 3.0 b-wave Peak time (ms)	N	37	37	37	37
	Median	30.5	31.5	1.0	3.3
	Min ; Max	28.0 ; 33.5	27.5 ; 38.5	-2.0 ; 7.0	-6.8 ; 22.2
	P5;P95	28.5;33.0	28.0;36.0	-1.5;3.0	-5.1;9.1
	Q1 ; Q3	30.0 ; 31.0	31.0 ; 33.0	0.0 ; 2.0	0.0 ; 6.5
	Mean ± SD	30.7 ± 1.3	31.7 ± 2.1	1.1 ± 1.5	3.4 ± 5.0
	SEM	0.2	0.3	0.3	0.8

		Table B1: Right eye (OD)			
Parameters	STAT	Baseline (M0)	M36A under treatment	M36A under treatment-Baseline (M0)	M36A under treatment-Baseline (M0)/Baseline (M0) x 100 $
LA 3.0 30 Hz flicker Peak time (ms)	N	37	37	37	37
	Median	27.0	29.0	0.5	1.9
	Min ; Max	25.0 ; 34.0	25.0 ; 35.0	-1.0 ; 7.0	-3.8 ; 28.0
	P5;P95	25.0;31.5	25.5;33.0	-1.0;4.0	-3.8;16.0
	Q1 ; Q3	26.5 ; 28.5	27.0 ; 30.0	0.0 ; 2.0	0.0 ; 7.3
	Mean ± SD	27.8 ± 1.9	28.9 ± 2.2	1.1 ± 1.6	4.0 ± 6.1
	SEM	0.3	0.4	0.3	1.0
BCVA (logMAR)	N	38	38	38	17
	Median	0.0	0.0	0.0	0.0
	Min ; Max	-0.2 ; 0.2	-0.2 ; 0.3	-0.2 ; 0.2	-200.0 ; 200.0
	P5;P95	-0.2;0.1	-0.2;0.2	-0.1;0.1	-200.0;200.0
	Q1 ; Q3	-0.1 ; 0.0	-0.1 ; 0.0	0.0 ; 0.0	-100.0 ; 0.0
	Mean ± SD	0.0 ± 0.1	0.0 ± 0.1	0.0 ± 0.1	-23.5 ± 95.4
	SEM	0.0	0.0	0.0	23.1
Color vision (TES)	N	38	38	38	31
	Median	28.0	30.0	0.0	0.0
	Min ; Max	0.0 ; 130.0	0.0 ; 194.0	-102.0 ; 166.0	-100.0 ; 866.7
	P5;P95	0.0;120.0	0.0;194.0	-84.0;132.0	-100.0;592.9
	Q1 ; Q3	16.0 ; 62.0	16.0 ; 78.0	-20.0 ; 34.0	-71.4 ; 109.5
	Mean ± SD	38.1 ± 36.1	48.8 ± 47.0	10.8 ± 54.3	57.3 ± 207.7
	SEM	5.9	7.6	8.8	37.3
VF Static (MD) (db)	N	35	35	35	34
	Median	1.5	1.5	0.1	-16.8
	Min ; Max	-1.5 ; 4.6	-1.5 ; 5.1	-2.9 ; 3.3	-475.0 ; 330.0
	P5;P95	-1.5;4.1	-0.9;4.7	-2.8;3.2	-216.7;266.7
	Q1 ; Q3	0.6 ; 2.9	0.2 ; 2.7	-0.6 ; 0.7	-74.4 ; 23.8
	Mean ± SD	1.6 ± 1.6	1.6 ± 1.7	0.0 ± 1.4	-14.3 ± 141.0
	SEM	0.3	0.3	0.2	24.2
VF Kinetic I3e (deg²)	N	36	36	36	36
	Median	6341.0	5617.5	-256.0	-4.0
	Min ; Max	3644.0 ; 8256.0	3488.0 ; 8817.0	-2727.0 ; 1014.0	-42.6 ; 13.9
	P5;P95	4000.0;8016.0	3659.0;8298.0	-2609.0;787.0	-38.2;12.2
	Q1 ; Q3	5243.5 ; 7225.5	4624.5 ; 6723.5	-985.5 ; 302.0	-15.6 ; 6.1
	Mean ± SD	6159.9 ± 1182.2	5727.8 ± 1413.8	-432.1 ± 992.5	-6.5 ± 15.4
	SEM	197.0	235.6	165.4	2.6
VF Kinetic III4e (deg²)	N	36	36	36	36
	Median	13133.0	13382.0	-268.5	-1.9
	Min ; Max	8606.0 ; 15604.0	8664.0 ; 15194.0	-2416.0 ; 2237.0	-18.8 ; 18.8
	P5;P95	10501.0;15217.0	10454.0;15172.0	-2239.0;1528.0	-15.4;11.4
	Q1 ; Q3	12348.0 ; 14380.5	12049.0 ; 14359.5	-606.5 ; 193.0	-4.5 ; 1.4
	Mean ± SD	13272.8 ± 1417.5	13098.6 ± 1536.5	-174.3 ± 896.6	-1.2 ± 6.9
	SEM	236.2	256.1	149.4	1.1

TABLE B2: Descriptive statistics for the baseline and M36 outcomes and for the differences over the 36 months: LEFT EYE

		Table B2: Left eye (OS)			
Parameters	STAT	Baseline (M0)	M36A under treatment	M36A under treatment-Baseline (M0)	M36A under treatment-Baseline (M0)/Baseline (M0) x 100 $
DA 0.01 b-wave Amplitude (µV)	N	38	38	38	38
	Median	197.9	193.8	-24.8	-14.3
	Min ; Max	120.8 ; 374.6	61.2 ; 431.6	-190.2 ; 182.3	-60.9 ; 119.4
	P5;P95	126.3;342.9	89.2;318.6	-175.8;171.8	-56.1;73.1
	Q1 ; Q3	154.4 ; 280.9	130.1 ; 230.8	-68.1 ; 8.0	-28.5 ; 2.6
	Mean ± SD	216.0 ± 72.0	191.3 ± 74.6	-24.6 ± 80.4	-6.9 ± 38.2
	SEM	11.7	12.1	13.1	6.2
DA 3.0 a-wave Amplitude (µV)	N	38	38	38	38
	Median	-177.4	-154.6	18.8	-9.5
	Min ; Max	-336.7 ; -77.9	-479.7 ; -49.7	-221.5 ; 116.4	-57.9 ; 109.1
	P5;P95	-319.1;-119.7	-280.6;-83.0	-144.8;100.1	-48.2;85.8
	Q1 ; Q3	-201.3 ; -150.1	-184.9 ; -137.8	1.3 ; 44.3	-28.0 ; -0.9
	Mean ± SD	-183.5 ± 52.3	-168.7 ± 72.7	14.8 ± 59.6	-7.4 ± 31.1
	SEM	8.5	11.8	9.7	5.1
DA 3.0 b-wave Amplitude (µV)	N	38	38	38	38
	Median	286.9	275.4	-24.2	-9.2
	Min ; Max	162.9 ; 489.9	93.8 ; 610.3	-20.7 ; 286.4	-55.8 ; 88.4
	P5;P95	196.5;479.1	155.1;489.1	-151.4;174.0	-46.8;71.2
	Q1 ; Q3	246.1 ; 341.0	204.6 ; 325.0	-66.0 ; 14.4	-20.2 ; 5.0
	Mean ± SD	303.4 ± 80.1	281.7 ± 99.3	-21.7 ± 85.4	-6.2 ± 27.1
	SEM	13.0	16.1	13.8	4.4
DA 12.0 a-wave Amplitude (µV)	N	38	38	38	38
	Median	-202.2	-192.5	17.8	-8.9
	Min ; Max	-450.1 ; -120.0	-558.6 ; -101.7	-212.7 ; 128.0	-48.3 ; 61.5
	P5;P95	-370.9;-146.2	-322.1;-109.0	-72.4;104.5	-44.0;39.7
	Q1 ; Q3	-234.8 ; -181.5	-224.0 ; -154.0	-2.4 ; 44.4	-21.8 ; 1.0
	Mean ± SD	-219.2 ± 64.3	-202.0 ± 77.9	17.2 ± 59.3	-7.1 ± 22.4
	SEM	10.4	12.6	9.6	3.6
DA 12.0 b-wave Amplitude (µV)	N	38	38	38	38
	Median	282.7	288.8	-23.0	-8.9
	Min ; Max	182.7 ; 532.5	110.5 ; 624.0	-227.6 ; 239.5	-56.9 ; 62.3
	P5;P95	184.3;518.1	172.5;503.7	-158.5;102.1	-40.0;44.3
	Q1 ; Q3	252.8 ; 358.6	222.2 ; 346.4	-60.8 ; 29.1	-21.2 ; 9.4
	Mean ± SD	312.1 ± 84.6	291.5 ± 96.1	-20.6 ± 80.5	-5.6 ± 23.0
	SEM	13.7	15.6	13.1	3.7
LA 3.0 a-wave Amplitude (µV)	N	38	38	38	38
	Median	-28.5	-24.0	3.4	-11.0
	Min ; Max	-57.6 ; -13.2	-51.6 ; -8.1	-21.8 ; 30.3	-53.3 ; 73.2
	P5;P95	-56.1;-17.6	-43.7;-10.7	-10.9;17.9	-52.6;43.7
	Q1 ; Q3	-32.8 ; -24.1	-29.7 ; -18.7	1.1 ; 7.1	-31.9 ; -2.9
	Mean ± SD	-29.2 ± 8.8	-25.3 ± 9.2	3.9 ± 9.0	-11.6 ± 27.4
	SEM	1.4	1.5	1.5	4.4
LA 3.0 b-wave Amplitude (µV)	N	38	38	38	38
	Median	107.1	93.3	-12.4	-13.9
	Min ; Max	42.6 ; 201.1	28.6 ; 168.7	-70.0 ; 57.7	-61.4 ; 65.2
	P5;P95	71.8;189.1	37.1;165.6	-61.6;41.7	-50.5;36.1
	Q1 ; Q3	93.7 ; 130.4	76.5 ; 113.1	-32.7 ; 7.0	-31.8 ; 7.0
	Mean ± SD	114.3 ± 32.6	99.1 ± 34.6	-15.2 ± 30.1	-11.9 ± 26.0
	SEM	5.3	5.6	4.9	4.2

		Table B2: Left eye (OS)			
Parameters	STAT	Baseline (M0)	M36A under treatment	M36A under treatment-Baseline (M0)	M36A under treatment-Baseline (M0)/Baseline (M0) x 100 $
LA 3.0 30 Hz flick. Amplitude (µV)	N	37	37	37	37
	Median	83.5	80.4	-6.7	-9.0
	Min ; Max	25.7 ; 156.0	22.1 ; 130.6	-58.9 ; 30.8	-58.6 ; 54.1
	P5;P95	46.8;146.9	28.8;119.1	-51.4;25.3	-45.6;49.2
	Q1 ; Q3	74.7 ; 107.8	70.3 ; 93.6	-27.5 ; 10.4	-29.2 ; 13.7
	Mean ± SD	91.0 ± 29.1	80.9 ± 23.7	-10.1 ± 22.9	-7.3 ± 26.0
	SEM	4.8	3.9	3.8	4.3
DA 0.01 b-wave Peak time (ms)	N	38	38	38	38
	Median	96.5	100.0	2.0	2.2
	Min ; Max	83.0 ; 119.0	75.5 ; 135.5	-20.5 ; 42.0	-21.4 ; 44.9
	P5;P95	85.0;115.5	84.0;126.0	-19.0;15.0	-17.7;17.6
	Q1 ; Q3	93.0 ; 102.0	95.0 ; 104.5	-1.0 ; 5.5	-1.1 ; 5.8
	Mean ± SD	97.5 ± 7.6	100.1 ± 10.9	2.6 ± 9.9	2.9 ± 10.3
	SEM	1.2	1.8	1.6	1.7
DA 3.0 a-wave Peak time (ms)	N	38	38	38	38
	Median	16.3	16.8	0.0	0.0
	Min ; Max	15.0 ; 18.5	14.5 ; 19.5	-1.5 ; 2.5	-8.8 ; 15.6
	P5;P95	15.0;18.5	15.5;18.5	-1.0;2.5	-6.5;14.7
	Q1 ; Q3	16.0 ; 17.0	16.0 ; 17.5	0.0 ; 0.5	0.0 ; 3.3
	Mean ± SD	16.5 ± 0.9	16.8 ± 1.1	0.3 ± 0.8	2.0 ± 5.1
	SEM	0.1	0.2	0.1	0.8
DA 3.0 b-wave Peak time (ms)	N	38	38	38	38
	Median	53.5	53.8	1.5	2.7
	Min ; Max	47.0 ; 59.5	47.0 ; 62.0	-8.0 ; 7.5	-13.4 ; 15.6
	P5;P95	48.0;58.0	47.0;60.0	-3.0;6.4	-6.0;13.6
	Q1 ; Q3	51.5 ; 55.5	52.4 ; 56.0	-1.5 ; 2.5	-2.7 ; 4.9
	Mean ± SD	53.3 ± 2.9	54.2 ± 3.3	0.9 ± 2.9	1.8 ± 5.6
	SEM	0.5	0.5	0.5	0.9
DA 12.0 a-wave Peak time (ms)	N	38	38	38	38
	Median	13.0	13.5	0.0	0.0
	Min ; Max	11.0 ; 15.5	10.5 ; 17.5	-2.5 ; 3.0	-17.9 ; 21.7
	P5;P95	11.5;15.0	11.0;15.0	-1.5;2.5	-12.5;20.7
	Q1 ; Q3	12.5 ; 14.0	12.0 ; 14.5	-0.5 ; 1.0	-3.6 ; 7.4
	Mean ± SD	13.1 ± 1.2	13.3 ± 1.4	0.2 ± 1.1	1.9 ± 8.6
	SEM	0.2	0.2	0.2	1.4
DA 12.0 b-wave Peak time (ms)	N	38	38	38	38
	Median	52.2	52.5	1.5	2.9
	Min ; Max	36.0 ; 60.0	41.0 ; 60.0	-11.5 ; 15.5	-21.9 ; 43.1
	P5;P95	40.5;57.5	45.5;59.5	-7.5;14.0	-13.6;34.6
	Q1 ; Q3	49.5 ; 54.5	51.0 ; 55.0	0.0 ; 4.5	0.0 ; 8.2
	Mean ± SD	51.1 ± 4.9	52.7 ± 3.9	1.6 ± 5.0	3.9 ± 11.3
	SEM	0.8	0.6	0.8	1.8
LA 3.0 a-wave Peak time (ms)	N	38	38	38	38
	Median	15.0	15.5	0.0	0.0
	Min ; Max	13.0 ; 17.0	11.5 ; 17.0	-5.5 ; 3.0	-32.4 ; 23.1
	P5;P95	13.5;17.0	13.5;17.0	-2.5;3.0	-15.6;22.2
	Q1 ; Q3	14.5 ; 16.0	14.5 ; 16.0	-0.5 ; 0.5	-3.1 ; 3.4
	Mean ± SD	15.1 ± 0.9	15.3 ± 1.1	0.2 ± 1.5	1.5 ± 10.0
	SEM	0.1	0.2	0.2	1.6
LA 3.0 b-wave Peak time (ms)	N	38	38	38	38
	Median	30.5	31.5	0.8	2.4
	Min ; Max	27.6 ; 33.5	27.5 ; 38.0	-1.5 ; 7.0	-5.2 ; 22.6
	P5;P95	28.0;33.5	27.5;37.0	-1.5;3.5	-4.5;10.4
	Q1 ; Q3	29.5 ; 31.5	30.5 ; 32.5	0.0 ; 1.5	0.0 ; 4.9
	Mean ± SD	30.5 ± 1.4	31.4 ± 2.1	0.9 ± 1.5	2.9 ± 5.0
	SEM	0.2	0.3	0.2	0.8

		Table B2: Left eye (OS)			
Parameters	STAT	Baseline (M0)	M36A under treatment	M36A under treatment-Baseline (M0)	M36A under treatment-Baseline (M0)/Baseline (M0) x 100 $
LA 3.0 30 Hz flicker Peak time (ms)	N	37	37	37	37
	Median	27.0	28.5	1.0	3.4
	Min ; Max	24.5 ; 31.0	24.5 ; 33.5	-2.5 ; 7.0	-8.2 ; 28.6
	P5;P95	24.5;31.0	25.0;31.5	-1.0;2.5	-3.8;9.8
	Q1 ; Q3	26.5 ; 29.0	27.0 ; 30.0	0.0 ; 2.0	0.0 ; 7.4
	Mean ± SD	27.6 ± 1.7	28.6 ± 2.1	0.9 ± 1.6	3.5 ± 6.2
	SEM	0.3	0.3	0.3	1.0
BCVA (logMAR)	N	38	38	38	21
	Median	0.0	0.0	0.0	0.0
	Min ; Max	-0.2 ; 0.2	-0.2 ; 0.3	-0.2 ; 0.2	-200.0 ; 200.0
	P5;P95	-0.1;0.2	-0.2;0.3	-0.1;0.2	-100.0;100.0
	Q1 ; Q3	-0.1 ; 0.0	-0.1 ; 0.0	0.0 ; 0.1	-100.0 ; 0.0
	Mean ± SD	0.0 ± 0.1	0.0 ± 0.1	0.0 ± 0.1	-9.5 ± 90.3
	SEM	0.0	0.0	0.0	19.7
Color vision (TES)	N	38	38	38	29
	Median	24.0	42.0	16.0	0.0
	Min ; Max	0.0 ; 174.0	0.0 ; 196.0	-142.0 ; 184.0	-100.0 ; 1533.3
	P5;P95	0.0;146.0	0.0;184.0	-100.0;132.0	-100.0;525.0
	Q1 ; Q3	12.0 ; 60.0	28.0 ; 58.0	-16.0 ; 42.0	-52.4 ; 175.0
	Mean ± SD	41.5 ± 45.9	55.6 ± 51.0	14.2 ± 64.0	101.5 ± 314.3
	SEM	7.4	8.3	10.4	58.4
VF Static (MD) (db)	N	33	33	33	30
	Median	1.7	1.1	-0.3	-34.6
	Min ; Max	-1.6 ; 6.3	-2.3 ; 5.4	-2.2 ; 2.4	-500.0 ; 314.3
	P5;P95	-0.9;4.9	-1.4;5.2	-2.2;2.2	-333.3;155.6
	Q1 ; Q3	0.0 ; 2.8	0.2 ; 2.8	-1.4 ; 0.8	-78.9 ; 16.7
	Mean ± SD	1.7 ± 1.9	1.5 ± 1.9	-0.2 ± 1.4	-39.5 ± 136.9
	SEM	0.3	0.3	0.2	25.0
VF Kinetic I3e (deg²)	N	37	37	37	37
	Median	6206.0	5531.0	-498.0	-7.3
	Min ; Max	3584.0 ; 8428.0	3332.0 ; 8593.0	-2758.0 ; 1335.0	-38.2 ; 31.0
	P5;P95	4179.0;8406.0	3629.0;8467.0	-2370.0;1111.0	-35.8;27.8
	Q1 ; Q3	5049.0 ; 7300.0	4695.0 ; 6575.0	-881.0 ; 22.0	-13.4 ; 0.5
	Mean ± SD	6237.8 ± 1353.8	5746.8 ± 1389.0	-491.0 ± 870.8	-7.1 ± 14.9
	SEM	222.6	228.4	143.2	2.5
VF Kinetic III4e (deg²)	N	37	37	37	37
	Median	13414.0	13271.0	-93.0	-0.8
	Min ; Max	9681.0 ; 16482.0	9807.0 ; 15735.0	-2689.0 ; 3122.0	-21.5 ; 32.2
	P5;P95	10277.0;15619.0	10050.0;15705.0	-1794.0;1664.0	-12.9;11.8
	Q1 ; Q3	12496.0 ; 14306.0	12137.0 ; 14706.0	-720.0 ; 560.0	-4.7 ; 3.9
	Mean ± SD	13321.8 ± 1510.7	13256.1 ± 1645.7	-65.7 ± 1065.5	-0.3 ± 8.8
	SEM	248.4	270.6	175.2	1.5

ANNEX C

Examples of differences over the 36 months for each participant by age at enrolment are presented graphically in Figure S1 for the LA 3.0 ERG b-wave amplitude (A) and peak time (B); the proportionate difference in the LA 3.0 30Hz flicker ERG (C); the Mean Defect visual field index (D); and the I3e (E) and the III4e isopter (F) areas. The direction of the red arrow illustrates the direction of the deterioration in function. The magnitude of the deterioration appeared to be independent of age at enrolment.

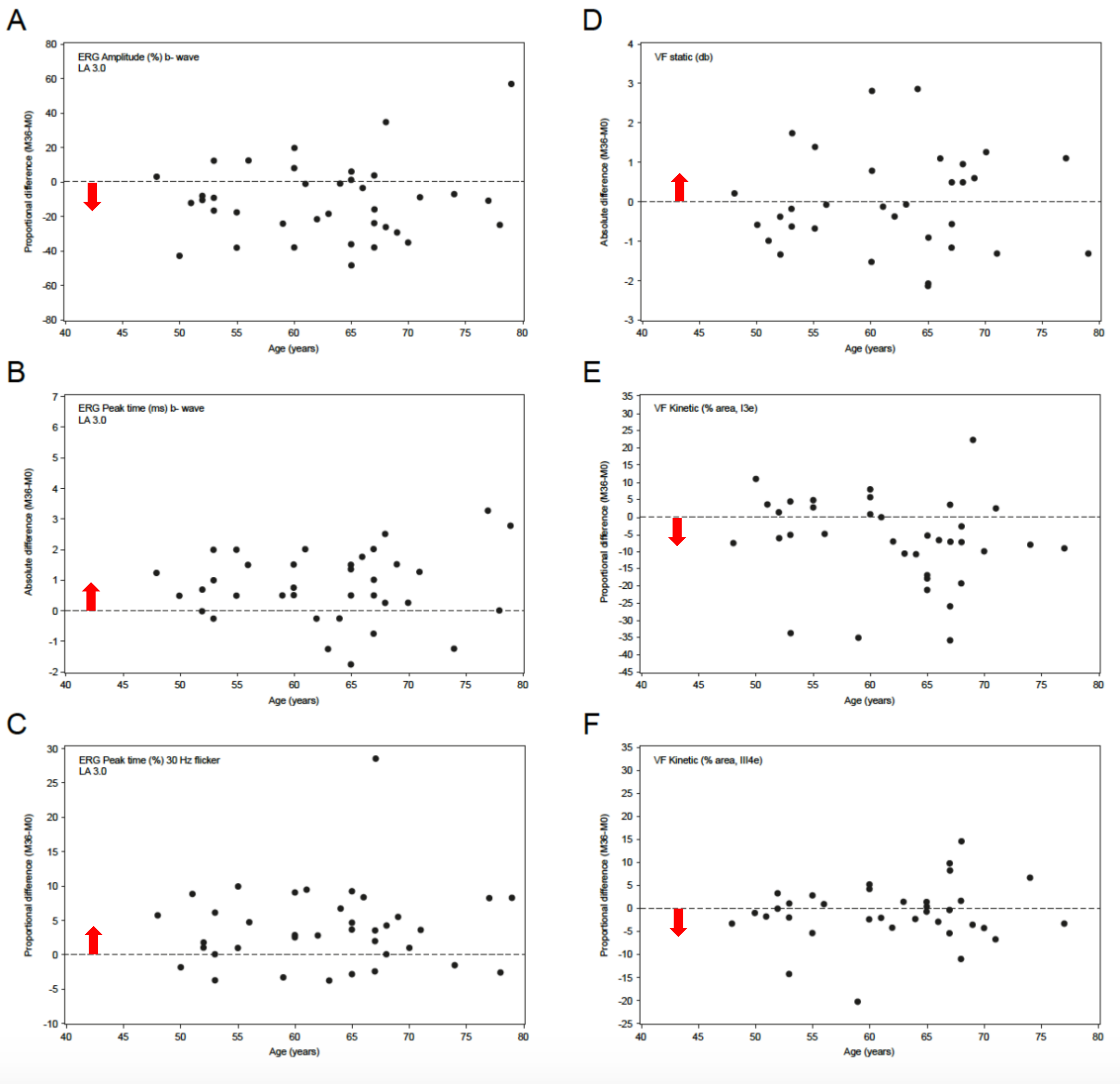

Fig. S1. The differences over 36 months by age at enrolment: the absolute difference in LA 3.0 ERG b-wave amplitude (A) and peak time (B); the proportionate differences in the LA 3.0 30Hz flicker ERG (C), the Mean Defect visual field index (D) and the I3e (E) and the III4e isopter (F) areas of SKP. In these examples, each data point represents a participant (data from left and right eyes were averaged for clarity). The red arrow indicates the direction of the worsening in performance.

ANNEX D

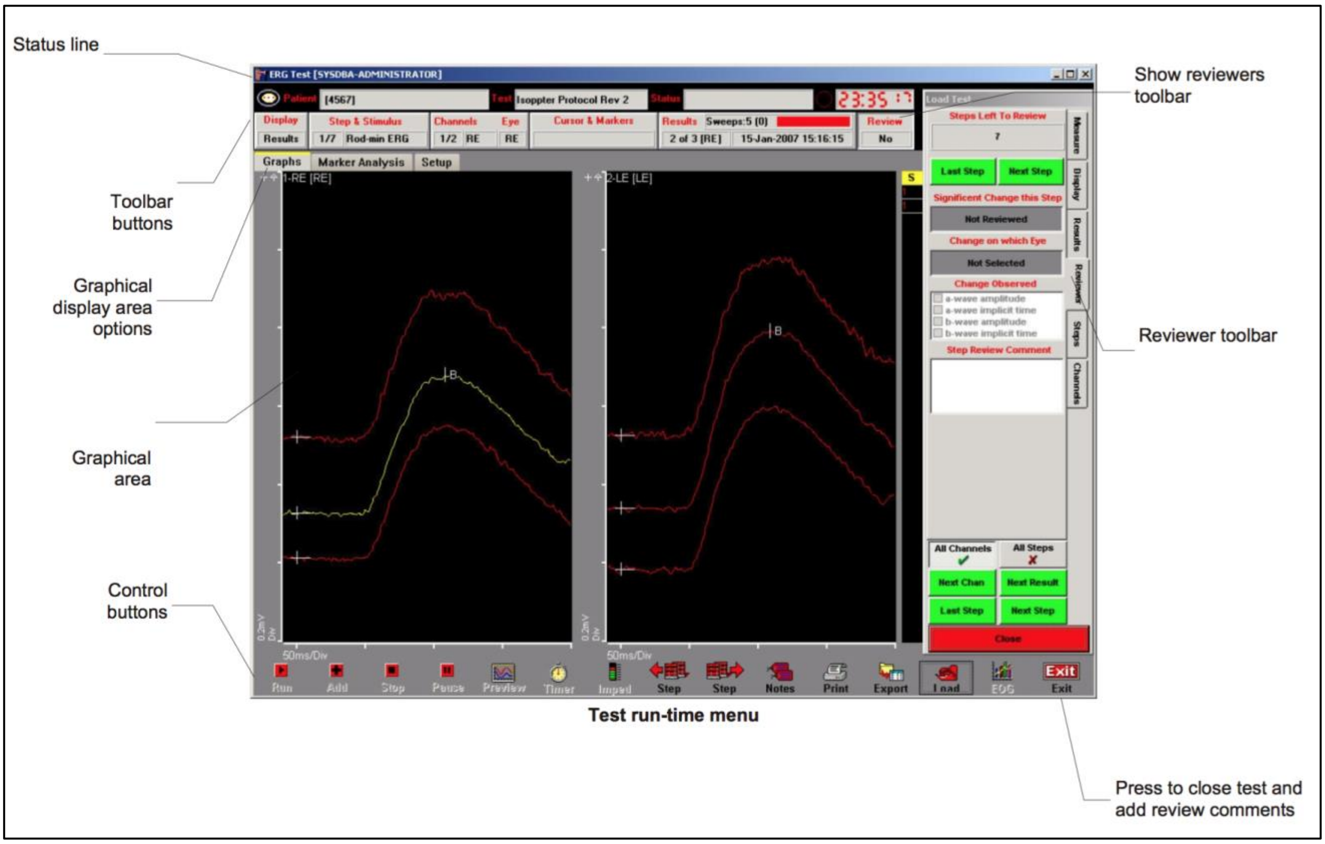

Fig S2. The WEB-based display of the DA 0.01 b-wave ERG viewed by the central reviewer. Three individual darkadapted rod responses are shown for both eyes (RE/LE) prior to averaging to a "result"for each eye separately. For further details see Zrenner et al.[26]
